# How do different components of Effortful Control contribute to children’s mathematics achievement?

**DOI:** 10.3389/fpsyg.2015.01383

**Published:** 2015-09-16

**Authors:** Noelia Sánchez-Pérez, Luis J. Fuentes, Violeta Pina, Jose A. López-López, Carmen González-Salinas

**Affiliations:** ^1^Department of Basic Psychology and Methodology, Faculty of Psychology, University of MurciaMurcia, Spain; ^2^Regional Campus of International Excellence “Campus Mare Nostrum”, University of MurciaMurcia, Spain; ^3^School of Social and Community Medicine, University of BristolBristol, UK; ^4^Department of Developmental and Educational Psychology, Faculty of Psychology, University of MurciaMurcia, Spain

**Keywords:** Effortful Control, attentional focusing, inhibitory control, mathematics achievement, childhood

## Abstract

This work sought to investigate the specific contribution of two different components of Effortful Control (EC) -attentional focusing (AF) and inhibitory control- to children’s mathematics achievement. The sample was composed of 142 children aged 9–12 year-old. EC components were measured through the Temperament in Middle Childhood Questionnaire (TMCQ; parent’s report); math achievement was measured *via* teacher’s report and through the standard Woodcock–Johnson test. Additionally, the contribution of other cognitive and socio-emotional processes was taken into account. Our results showed that only AF significantly contributed to the variance of children’s mathematics achievement; interestingly, mediational models showed that the relationship between effortful attentional self-regulation and mathematics achievement was mediated by academic peer popularity, as well as by intelligence and study skills. Results are discussed in the light of the current theories on the role of children’s self-regulation abilities in the context of school.

## Introduction

The importance of education related to science, technology, and engineering is increasing rapidly in industrialized societies (Sjøberg, 2002; Carnevale et al., 2011), with a consequent prominence given to mathematics and related disciplines in the different educational systems across countries. The acquisition of math abilities and more generally academic success has influential consequences on the students, as school performance has been associated with mental health (Bryant et al., 2000) as well as with future educational attainment (Marjoribanks, 2005), employment aspirations (Caspi et al., 1998), and socioeconomic position (Guglielmi, 2008). Given its relevance, it is important to identify which factors early influence mathematics achievement in order to be able to intervene and help students to perform better (Hintsanen et al., 2012). In this line, the present work aims to ascertain the contribution of temperamental self-regulation skills on the variance of mathematics performance in primary school children.

In addressing individual differences in school performance, research had traditionally focused on cognitive variables, with general intelligence as the most influential predictor of academic achievement (e.g., Neisser et al., 1996). More recently, a growing body of knowledge is highlighting that socio-emotional processes can explain an additional part of the variance of academic success (Valiente et al., 2007; Eisenberg et al., 2010; Rothbart, 2011; de la Fuente et al., 2014). Among these processes, children’s temperament, especially Effortful Control (EC), appears relevant to their school readiness and continued success in the academic domain (Eisenberg et al., 2010). From Rothbart’s psychobiological temperament framework, EC reflects individual differences in the efficiency of executive attention, and is defined as “the ability to inhibit a dominant response in order to perform a subdominant response, to detect errors, and to engage in planning” (Rothbart, 2011, p. 57). This voluntary control is considered a powerful moderator of cognitive, emotional, and behavioral processes.

There is an accumulated body of evidence showing concurrent and predictive relations between EC and academic achievement (e.g., Blair and Razza, 2007; Checa et al., 2008; Valiente et al., 2010, 2011, 2013; Zhou et al., 2010; Liew, 2012; Neuenschwander et al., 2012). Since EC is a multidimensional construct, the majority of studies have used an aggregated score that includes a set of dimensions representing the variety of processes covered by this temperament factor, but using a global score of EC does not permit to address the specific contribution of each component. As pointed out by Duckworth and Allred (2012, p. 639), “investigation is needed to establish which facets of EC are most important to academic success.”

Two components are key for EC: attentional focusing (AF), that is, the tendency to maintain attentional focus upon task-related channels, and inhibitory control (IC), or the capacity to both plan and suppress inappropriate approach responses under instructions. These processes reflect individual differences in the Executive Attentional Network, whose functioning involves the anterior cingulate gyrus and sections of the prefrontal cortex, and has close connections to adjacent motor systems (Vogt et al., 1992; Posner and Rothbart, 2000; Rothbart and Bates, 2006). Although correlated, these processes are dissociable and could differentially contribute to math achievement. In this work, we want to study separately both components of EC in order to gain a deeper understanding of the role that specific EC mechanisms might play in children’s mathematics achievement.

On one hand, AF involves individual differences in the ability to persist in ongoing tasks avoiding possible distractions, and should affect academic achievement because it influences children’s engagement in learning activities, completion tasks, and facilitates (or disrupts) classroom processes (Ladd et al., 1999; Pianta and Stuhlman, 2004; Posner and Rothbart, 2007). Previous research confirms that interpretation, as a higher attentional control in children and adolescents has been associated with better mathematics performance (Martin and Holbrook, 1985; Guerin et al., 1994; Gumora and Arsenio, 2002; Duncan et al., 2007; Checa et al., 2008; Rudasill et al., 2010; Hintsanen et al., 2012; Razza et al., 2012).

On the other hand, IC covers individual differences in the ability to suppress incorrect/inappropriate responses under instructions or in attending to social demands (Kochanska et al., 2000; Simonds and Rothbart, 2006). This self-regulatory ability involves the maintenance in working memory of a rule for correct responding while inhibiting a prepotent response tendency. In the school context, this ability would help to inhibit disrupting behaviors in the classroom, as well as to follow the instructions given by the teacher. As it also involves the capacity for planning and delay gratification (Mischel et al., 1989; Kochanska et al., 1997), it would help children in planning and using strategies when coping with school tasks as well as keeping in mind long-term objectives (Best and Miller, 2010; Eisenberg et al., 2010). In line with this interpretation, better inhibitory abilities have been associated with higher scores in children’s mathematics performance in preschool and kindergarten (Espy et al., 2004; Blair and Razza, 2007; Clark et al., 2010; Allan et al., 2014), as well as in elementary school ages (Welsh et al., 2010; Oberle and Schonert-Reichl, 2013).

As shown above, individual differences in AF and IC contribute to mathematics achievement. However, the relations generally account for a modest amount of variance, suggesting that other intellectual and socio-emotional aspects of the individuals are also important in explaining academic performance. Among the cognitive aspects, the relation between IQ and math performance has been extensively documented (Cronbach and Snow, 1977; Hunter and Hunter, 1984; Neisser et al., 1996; McGrew et al., 1997; Jensen, 1998; Keith, 1999; Taub et al., 2008; Alloway and Alloway, 2010) but additional explanatory dimensions related to children’s effort and proactive strategies such as academic-related skills, influence also math performance (Robbins et al., 2004; Credé and Kuncel, 2008).

At that respect, study skills comprise an array of coordinated cognitive skills and processes that enhance the effectiveness and efficiency of students’ learning that require a purposeful and conscious effort on the part of the student (Devine, 1987; Gettinger and Seibert, 2002). Research has already documented significant differences in both the quality and quantity of study strategies reported by high *versus* low achievers (e.g., Zimmerman and Martinez-Pons, 1990; Purdie and Hattie, 1996; Purdie et al., 1996; Ley and Young, 1998). Interestingly, these abilities explain a portion of the variance on academic achievement that is independent of intelligence (Gettinger and Seibert, 2002).

In the social sphere, peers become an important socialization source for pupils, especially in middle childhood and adolescence. Different aspects associated with the quality of peer relationships have been measured through several procedures during childhood and early adolescence, but results in connection to school achievement are in the same direction. For instance, Furrer and Skinner (2003) found that children’s self-reported sense of relatedness to peers correlated positively to overall academic achievement. The study by Oberle and Schonert-Reichl (2013) obtained a measure of peer acceptance informed by teachers, which was found positively associated with mathematics achievement. In the same line, Valiente et al. (2011) obtained a composite score reported by both teachers and parents that included children’s appropriate behaviors and popularity, and this measure was positively associated with grades. Explanations for these associations have focused on the notion that belonging to a friendship group in school can increase motivation to engage in school activities and be a valuable source of social support for students in the school context (Furrer and Skinner, 2003; Oberle and Schonert-Reichl, 2013). Another strategy, the peer-nomination technique, allows obtaining an index of children’s social preference in work situations and leisure time at school. Although many studies have used a global measure of social preference (e.g., Wentzel, 1991; Wentzel and Caldwell, 1997; Ladd et al., 1999; Welsh et al., 2001; Ladd, 2003), popularity in the context of schoolwork *versus* leisure time could be especially relevant to academic achievement in the last years of primary school. Adopting Vygotsky’s (1978) social constructivism, children interacting in cooperation would benefit from conversations in working with their peers when dealing with school tasks; in this way, they would exchange ideas and receive information, thereby generating understanding and developing knowledge. Although research considering academic *versus* leisure contexts at school is scarce, the work by Checa et al. (2008) points to the relevance of academic over leisure preference in explaining mathematics achievement in a sample of secondary school students.

In addition, the aforementioned factors have proved interrelated, as EC has been associated with intelligence and executive functions (Rothbart et al., 2007; Simonds et al., 2007; Zhou et al., 2012), academic competences (Checa et al., 2008; Valiente et al., 2013), and social skills and popularity (Fabes et al., 1999; Eisenberg et al., 2000; Spinrad et al., 2006; Checa et al., 2008; Valiente et al., 2011). Bearing in mind these interactions, we regard the temperament-academic achievement association as complex, with multiple mechanisms likely involved including cognitive, motivational, and interpersonal ones (Rothbart and Jones, 1998). Nonetheless, only recently investigators have begun to specify the pathways and mechanisms through which EC influences academic achievement (Valiente et al., 2011). In the present study, we aim to test two pathways through which EC components would contribute to mathematics performance in primary school children.

The first pathway, proposed by Eisenberg et al. (2005), specifies that the association between children’s EC and academic performance may be partly mediated by children’s social competences. Although research testing this contention is still scarce, some studies point out that the association EC-academic achievement is partially mediated by children’s social competence, peer acceptance, classroom participation, teacher–child relationship, and school liking (e.g., Valiente et al., 2007, 2008, 2011; Zhou et al., 2010; Oberle and Schonert-Reichl, 2013). Consistently with such evidence, we hypothesize that children with higher AF and IC will be more popular amongst their peers in the context of schoolwork, which in turn would constitute the social resources for mathematical learning.

The second pathway is based on the premise that EC mechanisms interact with cognitive processing in the school context (Rothbart and Jones, 1998), and individual differences in self-regulation abilities would influence higher order cognitive processes such as fluid intelligence. As previously mentioned, EC depends on the Executive Attention Network, which in turn is considered the psychological core of the psychometric construct of fluid intelligence (Kane and Engle, 2002). Moreover, studies on training executive attention have reported transferred effects to fluid intelligence (Rueda et al., 2012), supporting the notion that there is an extended overlap between the brain structures implicated in general intelligence and those of the Executive Attention Network (Duncan et al., 2000). Following this line of research, we hypothesize a mediation model in which the attentional and inhibitory components of EC would be involved in the acquisition of general cognitive skills (IQ), as well as more specific academic-related skills (study skills), which in turn would affect children’s math achievement. As far as we know, there is no previous study showing a mediational pathway of EC on math achievement involving intelligence. Only Neuenschwander et al. (2012) have tested a mediational role of “learning-related behaviors” – a measure that included persistence, efficiency of homework, and self-reliance in coping with school tasks-, in the relationship between EC and academic achievement in a sample of kindergarten and early elementary pupils.

The proposed models may be especially relevant in explaining children’s mathematics achievement in the last years of primary school because of the mediational processes involved. Concerning academic abilities, study skills are specifically instructed and are part of the strategies children should use in learning in this last period of elementary school. These strategies involve the acquisition, organization and retention of information in an intentional and purposeful way, and require self-regulatory behaviors such as initiative, persistence, or goal setting (Gettinger and Seibert, 2002); the efficiency of its use largely depends on metacognition and control processes. From a developmental point of view, middle childhood is considered crucial for the development of metacognitive monitoring and study of control processes (Metcalfe and Finn, 2013). With respect to academic peer popularity, is during middle childhood and early adolescence that the social focus shifts away from the family and toward the peer group (Larson and Richards, 1991), and being accepted and having friends at school emerges as an important aspect for positive growth in school (Oberle and Schonert-Reichl, 2013).

Additionally, two other variables have been considered in this study: gender and socioeconomic status (SES). Some studies addressing the effect of gender in mathematics achievement have not found differences between boys and girls (Davis-Kean, 2005; Simpkins et al., 2006; Matthews et al., 2009), while others have found that boys outperformed girls on math abilities (Frome and Eccles, 1998; Jordan et al., 2006; Else-Quest et al., 2010; Hintsanen et al., 2012; Instituto de Evaluación, 2014). Due to the inconsistency of findings, more research is clearly needed at this respect.

Lastly, SES has been included in our study because previous research suggests that parents’ location in the socioeconomic structure has a strong impact on students’ academic achievement (Hart and Risley, 1995; Hoff-Ginsberg and Tardif, 1995; McLoyd’s, 1998; Kohl et al., 2000; Davis-Kean, 2005; Dubow et al., 2009; Aunio and Niemivirta, 2010; Valiente et al., 2011; Carvalho and Novo, 2012; see Sirin, 2005 for a meta-analytic review). Explanations for this relationship point that SES positively affects parents involvement in their children’s education (Kohl et al., 2000), the quality of parenting (McLoyd’s, 1998) as well as parent’s expectations and children’s educational aspirations (Davis-Kean and Schnabel, 1999; Dubow et al., 2009). Nevertheless, this association has varied depending on the SES factors and the school subject studied. For instance, Davis-Kean and Schnabel (1999) found that different indices of SES proved positively associated with reading and math achievement in a sample of children aged 8–12 years. However, mother’s education did not contribute significantly to reading achievement, whereas the status of the mother as working out of home predicted negatively children’s maths achievement. More recently, Aunio and Niemivirta (2010) proved a positive relation of SES to arithmetic abilities as measured through a standard test, but this was not true for a global measurement of academic achievement or for children’s grades. Given the current situation, we want to test the contribution of SES on children’s mathematics achievement.

In summary, the aim of this work is to test two pathways through which EC components would contribute to mathematics performance in primary school children, as measured through a standard test and *via* teacher’s report. In a relational pathway, academic peer popularity would mediate the association EC-mathematics achievement; in the intellectual-abilities pathway model, the mediational factors proposed are non-verbal intelligence and study skills. The role of gender and SES is also considered in this study.

## Materials and Methods

### Participants

One hundred and forty-two children (68 boys), their parents and teachers participated in this study. Children aged between 9 and 12 years (*M* = 10.5 years, *SD* = 0.96 years) and had no diagnosis of any learning disability or clinical disorder. This sample was recruited from eight urban and suburban schools in the Region of Murcia (SE, Spain), which specifically consented to participate in this study.

According to information provided by the parents and regarding the ethnic background, 91.5% of the children were European, 4.3% Latin American, 3.5% African, and 0.7% Gypsy, representing the ethnic variability of the geographical area. Children came predominantly from two-parent homes (90.4%). In terms of parental education, 53.5% of the mothers (percentages for fathers are in parentheses; 53.3%) were educated at the elementary school level, 23.6% (25.5%) at high school level, and 22.9% (21.2%) at university level. Concerning monthly incomes, 9.9% reported to receive less than 750€ (lower extreme compared to the average family income^1^), 24.3% reported from 751 to 1200 (well below average), 21.6% from 1201 to 1600 (below average), 8.2% from 1601 to 2000 (in average), 23.4% from 2001 to 3000€ (above average), and 12.6% parents reported more than 3000€ (well above average) as monthly family income.

This study involved a total of 31 teachers (seven males, 24 females) aged between 24 and 60 years (*M* = 47.8 years, *SD* = 11.2 years). Teachers provided information about those students who participated in the research, between 2 and 14 children per class. They were not paid for their contribution.

### Measures

#### Mathematics Achievement

Most studies addressing children’s academic performance have relied on a unique kind of measurement; they have opted for using standardized achievement tests (e.g., Martin et al., 1988; Birch and Ladd, 1997; Howse et al., 2003; Blair and Razza, 2007; Valiente et al., 2010) or teacher’s reports (e.g., Zhou et al., 2010; Valiente et al., 2011, 2013; Hintsanen et al., 2012) as a measure of long-term retention of learned information. Every kind of measurement has its own advantages and limitations; standardized tests allow researchers to assess the level of acquisition of specific school abilities and to compare children’s scores across ages and grades. However, it can have limitations to cover full knowledge of the children (DuPaul et al., 1991). In contrast, teacher’s reports provide a comprehensive sample of academic content, including information about children’s acquisition of the school subjects as well as student’s behavior in the classroom. Nevertheless, teachers could also introduce a certain bias in their reports (Pullis, 1985; Martin, 1989; Keogh, 2003). Since standard assessments and teacher’s reports cannot be considered equivalent measurements and each one has its own limitations, we have included both kinds of measures in our study to gain a more comprehensive assessment of children’s mathematics performance.

##### Mathematics achievement: standard test

Children’s mathematics abilities were measured through the *Calculation* and *Applied Problems* subtests of the Woodcock Johnson III Tests of Achievement (WJ-III; Woodcock et al., 2001; Spanish version by Muñoz-Sandoval et al., 2005), validated for their use in Spain by Diamantopoulou et al., 2012). *Calculation* assesses the ability to make mathematical calculations, and *Applied Problems* evaluates the ability to analyze and solve mathematical problems. The scoring for every item is 0 (*incorrect*) and 1 (*correct*). Direct scores of Calculation and Applied Problems were computed as the sum of correct answers. These scales were moderately correlated in this sample (*r* = 0.40; *p* < 0.001). The final score used was short-W provided by the WJ III Normative Update Compuscore and Profiles Program (Schrank and Woodcock, 2007), which includes *Calculation* and *Applied Problems* subtests. Short W score is obtained applying a Rasch transformation on the direct measures of each subtest and lastly averaging both subscales.

##### Mathematics achievement: teacher’s report

Teachers completed a short questionnaire with three items about children’s mathematics achievement. They scored children’s level of math acquisition in comparison to their classmates. The items covered the following math abilities: “Solving numerical problems,” “working with forms (e.g., geometry)” and “understanding mathematical measures.” Children’s math abilities were scored using a five-point Likert-scale rating (1 = well below the average, 2 = below the average, 3 = in average, 4 = above the average, 5 = well above the average). Cronbach’s alpha for this questionnaire in our sample was 0.96. A global measure of mathematics achievement was calculated averaging the score given to each of the three items.

##### Attentional and inhibitory mechanisms of EC

Parents completed a Spanish version of the Temperament in Middle Childhood Questionnaire (TMCQ; Simonds and Rothbart, 2006), translated and back-translated with permission of the authors. Parents had to evaluate the extent to which each statement properly described his/her child’s behavior within the previous 6 months. The scale ranged from 1 (almost always untrue) to 5 (almost always true), with an additional option of “Not applicable.” TMCQ assesses 17 lower-order facets of temperament. For the purpose of this study, we selected the items included in AF and IC scales. Cronbach’s alpha for AF in this sample was 0.86 but IC brought out an unsatisfactory index of 0.55. Then, item-test correlation was examined for every item of this scale and it was found out that the item “has an easy time waiting to open a present” showed a corrected item-test correlation of 0.05. Once this item was excluded, the resulting coefficient was α = 0.61, with an average item-test correlation of 0.32. The score in each scale was calculated dividing the total by the number of items receiving a numerical response. AF and IC correlated each other positively with a Pearson coefficient of *ρ* = 0.37 (*p* < 0.001). This magnitude informs that although correlated, each scale covers rather different mechanisms of EC.

##### Non-verbal intelligence

Children’s non-verbal intelligence was measured using a Spanish version of the Matrices subtest of the Kaufman Brief Intelligence Test (K-BIT; Kaufman and Kaufman, 1990). The reliability of the Matrices subtest for the Spanish version measured as Alpha coefficient ranged from α = 0.87 to α = 0.93 for the ages 9–12 years (Kaufman and Kaufman, 1990). The total score was the sum of correct responses.

##### Study skills

Teachers reported children’s learning-related abilities using a Spanish version (González et al., 2004) of the study skills scale from the Behavior Assessment System for Children (BASC; Reynolds and Kamphaus, 2004). Teachers were asked to identify the frequency, ranged from 1 (never) to 4 (almost always), with which children showed a specific behavior related to any academic competence. The study skills scale covers behaviors and skills that lead to high academic performance. It includes behaviors such as reading or studying that are oriented to learning (e.g., uses the school’s library); organized strategies in coping with academic tasks (e.g., analyzes a problem carefully before solving it); hard work and effort (e.g., strives even in the subjects s/he does not like), and self-confidence in undertaking academic tasks (appears confident in coping with exams). Cronbach’s alpha for this scale in our sample was 0.92. A total score was obtained by averaging the scale items.

##### Academic peer popularity

A peer nomination of children’s sociability-leadership was obtained using a sociometric questionnaire. Children chose three classmates whom they would like to form a group with to do the schoolwork or to study (election) and three classmates whom they would not (rejection). For each child, an election index was obtained from the number of choices received divided by the number of children participating minus one and multiplied by 100 (Bezanilla, 2011). Rejection index was obtained using the same procedure. This formula permits to compare the sociometric status of children belonging to different size groups. Peer popularity was the result of the choice index minus the rejection index.

##### Socioeconomic status (SES)

An index of SES was obtained for each child taking into account three variables: (1) mother’s years of schooling; (2) father’s years of schooling; and (3) monthly family incomes. Each variable was standardized and then averaged in order to form a composite score of SES.

Means and SD for the variables under study are provided en **Table 1**.

**Table 1:** Means and SD (in parentheses) for the variables under study.

Math achievement							
Standard test	Teacher’s report	Attentional focusing (AF)	Inhibitory control (IC)	IQ	Study skills	Academic popularity	SES
501.40 (10.58)	3.25 (0.98)	3.22 (0.86)	3.6 (0.54)	26.81 (4.50)	2.63 (0.74)	6.25 (22.42)	-0.02 (0.86)

### Procedures

The study was approved by the Ethics Committee of the University of Murcia. Children and parents had previously participated in a larger study to validate the four mathematics tests of the Woodcock–Johnson III Achievement battery (*N* = 424) in a Spanish sample (Diamantopoulou et al., 2012). To obtain a sample representative of the normal population of children, we selected eight urban and suburban schools in low-, medium-, and high SES residential areas.

In the next academic year, and with the aim of analyzing the contribution of cognitive and socio-emotional factors to math achievement, we requested again permission from those families whose children were enrolled between 4th and 6th grade (*N* = 156). For this study, parent information letters describing the research project and consent forms were delivered to the participant families. The return rate was 91% (*N* = 142). Parents who agreed to participate also received at home the temperament questionnaire with instructions to complete it. Once completed, parents returned it to school. A person from our research team was available at school for attending any question raised by the parents. Teachers were asked to complete a questionnaire referred to children’s study skills as well as to mathematics achievement.

All the variables were measured in the second term of the academic year (January–April 2012). Trained assistants at school assessed children’s math abilities and intelligence. A small room assigned by the head-teacher from each school permitted to administer both tests individually in one session lasting from 30 to 45 min. Tests were administered in a counterbalanced sequence to control for systematic variations due to order of administration. At the end of the session, children received a small prize for their participation.

The sociogram^2^ was administered by a member of our staff in the classroom in the presence of the tutors. All the classmates completed the questionnaire, but only information concerning the children who participated in this study was taken into account.

### Data Transformation

In order to control for the influence of age, all variables to be included as mediators or dependent variables in the mediational analyses were standardized by grade.

## Results

### Bivariate Analyses

Analyses were conducted in SPSS (version 19) using listwise to deal with missing values. We first computed a series of preliminary analyses to test for potential school and gender differences. An analysis of variance (ANOVA) was run taking School as the independent variable and scores in the Woodcock–Johnson Battery as the criterion. Gender effect on mathematics achievement was tested using *t*-test analyses for independent groups. Since no significant differences were found, School and Gender were not further included in the data analyses.

To test the relations of the attentional and inhibitory mechanisms of EC with the dependent and mediational variables, we computed zero-order correlations for AF, IC, math achievement, SES, study skills, IQ, and academic popularity. As seen in **Table 2**, AF correlated moderately with the two dependent variables but IC correlations did not reach statistical significance. This is why IC was not further included in the mediational analyses. Academic popularity, IQ, and study skills could be considered as mediational because they correlated significantly with both AF and mathematics achievement variables, with an *r*-value above 0.20.

**Table 2:** Zero-Order correlations for the study variables.

	Variables	1	2	3	4	5	6	7	8
1	Math achievement (standard test)	-							
2	Math achievement (teacher’s report)	0.55^∗∗∗^	-						
3	AF	0.22^∗∗^	0.27^∗∗^	-					
4	IC	-0.03	0.01	0.37^∗∗∗^	-				
5	IQ	0.48^∗∗∗^	0.36^∗∗∗^	0.22^∗∗^	0.03	-			
6	Study skills	0.41^∗∗∗^	0.59^∗∗∗^	0.55^∗∗∗^	0.27^∗∗^	0.34^∗∗∗^	-		
7	Academic peer popularity	0.24^∗∗^	0.37^∗∗∗^	0.37^∗∗∗^	0.15	0.04	0.58^∗∗∗^	-	
8	Socioeconomic status (SES)	0.24^∗∗^	0.20^∗^	0.16	-0.05	0.37^∗∗∗^	0.20^∗^	0.00	-

*^∗^*p* < 0.05; ^∗∗^*p* < 0.01; ^∗∗∗^*p* < 0.001.*

All the variables considered in the mediational analyses were tested for the normality assumption. In addition, given the nested nature of our data set, we computed intraclass correlations with school as the grouping factor. For all the variables included in our analyses, we obtained (absolute) correlations below 0.1, with their confidence intervals always containing zero. Therefore, children’ scores were treated as independent, and hence analyses from the classical linear model were conducted.

### Mediational Analyses

In the next set of analyses, we tested both a relational and an intellectual pathway model in which academic peer popularity on the one hand, and non-verbal IQ and study skills on the other hand, would mediate the relation between AF and math achievement (standard test and teacher’s report separately). Mediation was tested following the procedures described in Baron and Kenny (1986). We also conducted the bootstrapping procedure using the SPSS macros provided by Preacher and Hayes (2008) to verify the indirect effects on the mediational models. A 95% confidence interval was used to measure mediation (Preacher and Hayes, 2008; Zhao et al., 2010).

#### The Relational Model Testing Academic Peer Popularity as Mediator in the Attentional Focusing and Math Achievement (Standard Test) Association

**FIGURE 1:**
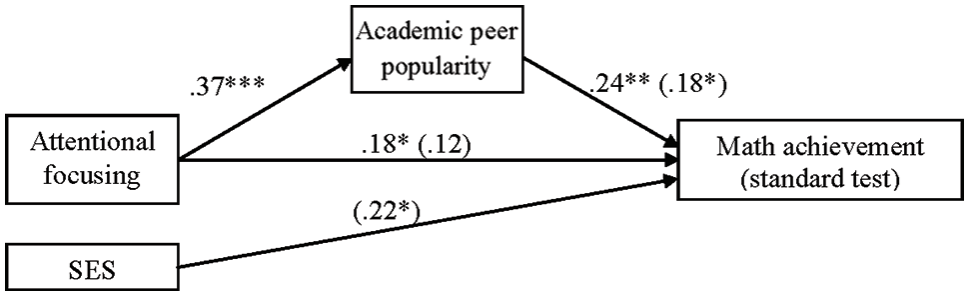
**Relational model with academic academic peer popularity as mediator in the attentional focusing (AF) and math achievement (standard test) association.** Numbers in the figure are beta coefficients (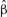 for mediational model are in parentheses). ^∗^*p* < 0.05; ^∗∗^*p* < 0.01; ^∗∗∗^*p* < 0.001.

As explained above, we firstly examined the relational pathway model with the mathematics standard test score as the dependent variable. First, we tested whether academic peer popularity would partially mediate the relation between AF and math achievement (standard test and teacher’s report), taking into account the potential effect of SES on the dependent variable. Regressing academic peer popularity on AF yielded a significant effect [*F*(1,140) = 21.73, *p* < 0.001, *R^2^*_adj_ = 0.13]. AF was a significant positive contributor of social preference in the context of the classroom (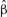 = 0.37, *p* < 0.001). Thus, children with higher attentional skills were more preferred among their classmates in undertaking academic activities. For the second equation of mediation, math achievement (standard test) was regressed on AF yielding a significant effect [*F*(2,131) = 6.46, *p* = 0.002, *R*^2^_adj_ = 0.08]; then, AF was a significant positive contributor of math achievement (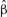 = 0.18, *p* = 0.032), even after controlling the effect of SES (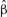 = 0.21, *p* = 0.014). In the last step of mediation, regressing math achievement (standard test) on AF and popularity yielded a significant effect [*F*(3,130) = 5.75, *p* = 0.001, *R*^2^_adj_ = 0.10]. This model explained 10% of variance on standard math achievement. Academic peer popularity (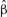 = 0.18, *p* = 0.047) exerted a significant positive influence on math achievement, whereas the relation between AF and the dependent variable was non-significant (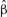 = 0.12, *p* = 0.204), after taking into account the contribution of SES (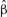 = 0.22, *p* = 0.010; see **Figure 1**). Children who were more popular among their classmates in addressing academic activities, showed higher scores on the standard test of math achievement; as the mediational model was proved, we can infer that children’s attentional control had a positive influence on academic peer popularity, which in turn had an impact on math achievement’ scores measured *via* the standard test.

We tested this model with the bootstrapping technique, in which the confidence interval did not include zero (range = 0.01–0.17); thus, mediation was established.

#### The Relational Model Testing Academic Peer Popularity as Mediator in the Attentional Focusing and Math Achievement (Teacher’s Report) Association

With respect to the relational pathway model applied to teacher’s mathematics report as dependent variable, the first mediational equation was the same as in the previous analyses with the standard test. In the second step of mediation, teacher’s report was regressed on AF showing a significant effect [*F*(2,129) = 6.62, *p* = 0.002, *R*^2^_adj_ = 0.08]; then, AF was a significant positive contributor of math achievement informed by teacher’s report (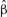 = 0.23, *p* = 0.007), after controlling the effect of SES (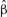 = 0.16, *p* = 0.06). Children with better attentional control obtained higher scores on math achievement as reported by their teachers.

**FIGURE 2:**
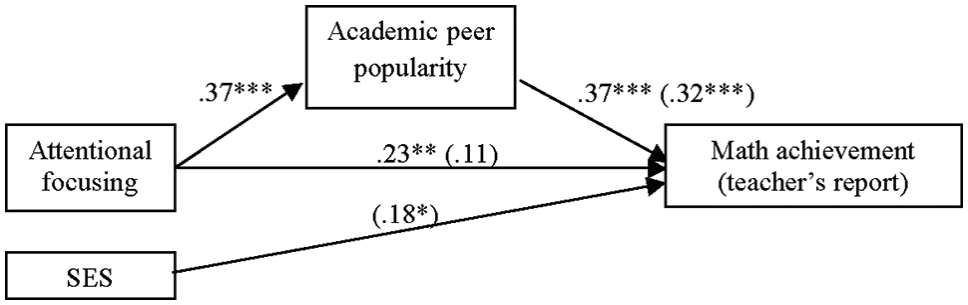
**Relational model with academic peer popularity as mediator in the AF and math achievement (teacher’s report) association.** Numbers in the figure are beta coefficients (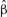 for mediational model are in parentheses). ^∗^*p* < 0.05; ^∗∗^*p* < 0.01; ^∗∗∗^*p* < 0.001.

In the last equation, regressing math achievement on AF and academic popularity yielded a significant relationship [*F*(3,128) = 9.57, *p* < 0.001, *R*^2^_adj_ = 0.16], with a 16% of explained variance on teacher’s math report. After controlling the effect of SES (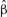 = 0.18, *p* = 0.030), children’s peer popularity (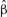 = 0.32, *p* < 0.001) exerted a significant positive influence on math achievement, whereas the relation between the independent variable (AF) and the dependent variable was now non-significant (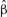 = 0.11, *p* = 0.21; see **Figure 2**). As the previous analyses showed, children who were more popular academically obtained higher scores on math achievement reported by their teachers; also, children’s academic popularity mediated in the relation between attentional skills and math achievement reported by teachers. Bootstrapping test did not include zero in the confidence interval (range = 0.07–0.24) and mediation was established.

#### The Intellectual Abilities Model Testing IQ and Study Skills as Mediators in the Attentional Focusing and Math Achievement (Standard Test) Association

To test the intellectual-abilities pathway model, we used the aforementioned procedures. In the first step, regressing IQ on AF yielded a significant effect [*F*(1,139) = 7.19, *p* = 0.008, *R*^2^_adj_ = 0.04]. AF was a significant positive predictor of IQ (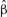 = 0.22, *p* = 0.008). Study skills on AF yielded a significant effect [*F*(1,137) = 58.85, *p* < 0.001, *R*^2^_adj_ = 0.30]. AF was a significant positive contributor of study skills (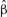 = 0.55, *p* < 0.001). Then, children with higher attentional control obtained higher scores on IQ and better study skills. The second step was the same as in the first model, where AF yielded a significant effect on math achievement (standard test; 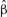 = 0.18, *p* = 0.032).

**FIGURE 3:**
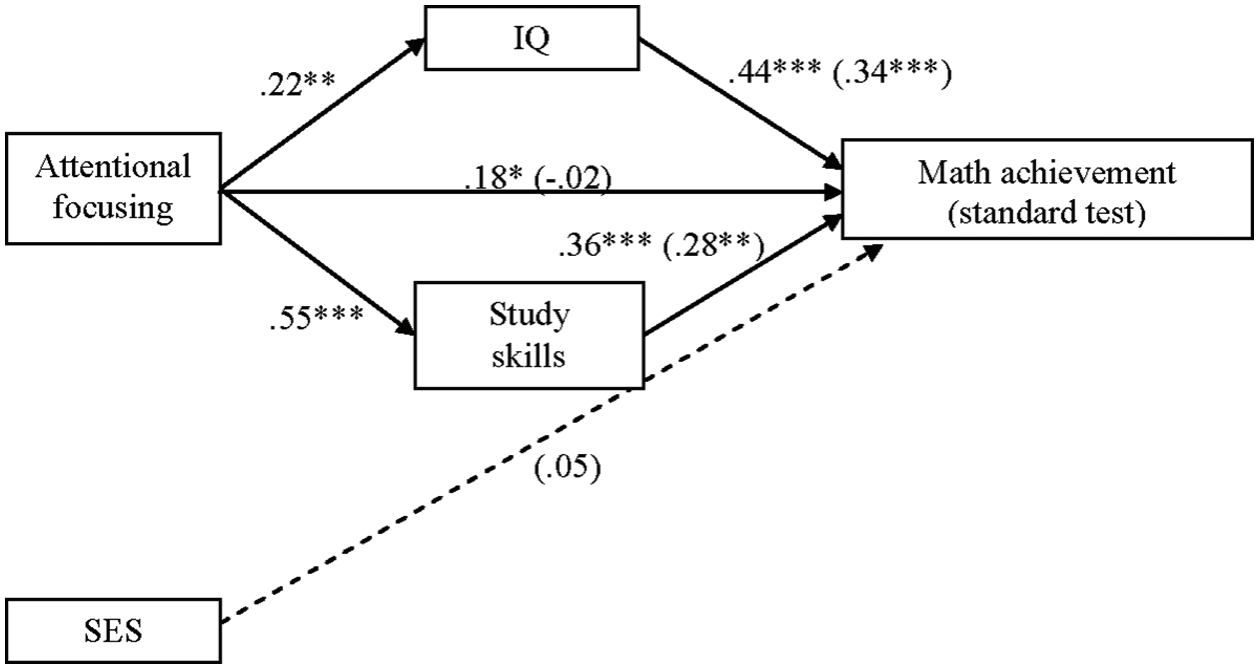
**Intellectual-abilities model with IQ and study skills as mediators in the AF and math achievement (standard test) association.** Numbers in the figure are beta coefficients (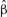 for mediational model are in parentheses). ^∗^*p* < 0.05; ^∗∗^*p* < 0.01; ^∗∗∗^*p* < 0.001.

In the last equation, the relationship between AF and math achievement (standard test) was tested again, with the influence of each potential mediator controlled for (IQ and study skills). Regressing math achievement on AF, study skills, and IQ yielded a significant effect [*F*(4,126) = 11.79, *p* < 0.001, *R*^2^_adj_ = 0.25]. This model explained a 25% of variance on standard math achievement. Study skills (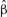 = 0.28, *p* = 0.003) and IQ (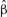 = 0.34, *p* < 0.001) both exerted a significant direct influence on math achievement, even after controlling for the effect of SES (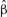 = 0.05, *p* = 0.54), whereas the relation between AF and the criterion was non-significant (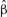 = -0.02, *p* = 0.85; see **Figure 3**). Thus, children with higher scores on IQ and better study skills obtained better scores on the standard test of math achievement and, as we hypothesized, children’s cognitive processes acted as mediators in the relation between AF and math achievement (standard test). We tested this model with the bootstrapping technique, where the confidence interval for IQ (range = 0.03–0.17) and Study skills (range = 0.06–0.32) did not include zero; then, mediation was established.

#### The Intellectual Abilities Model Testing IQ and Study Skills as Mediators in the Attentional Focusing and Math Achievement (Teacher’s Report) Association

**FIGURE 4:**
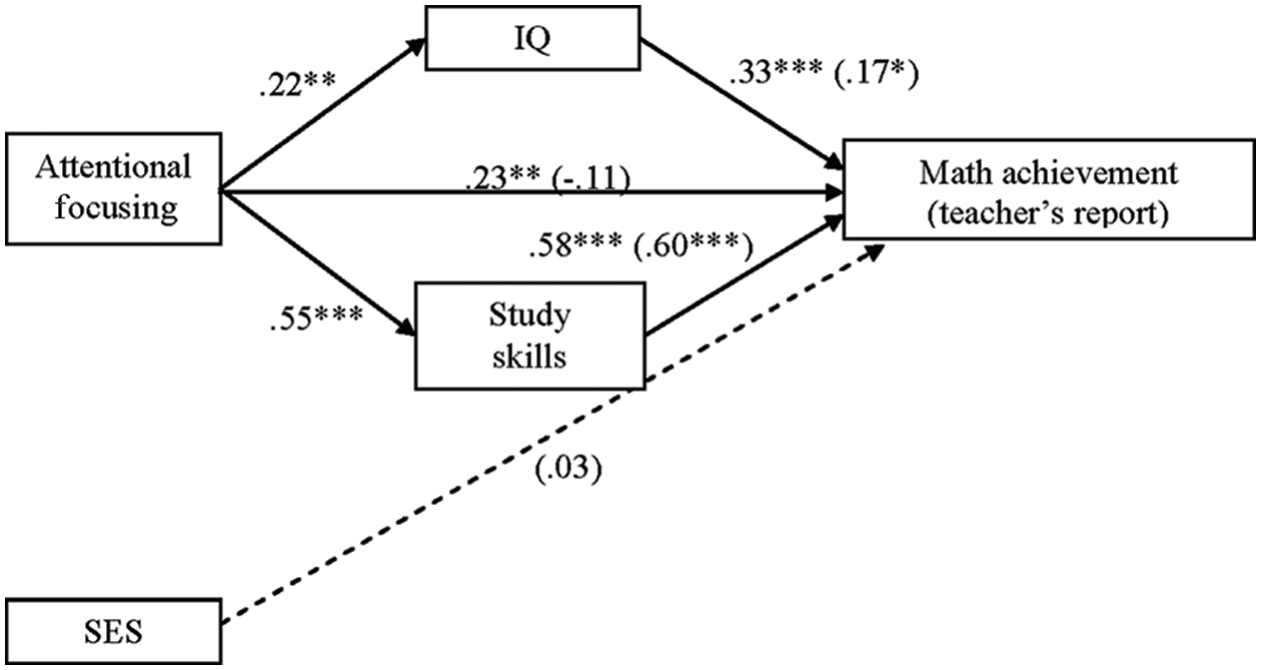
**Intellectual-abilities model with IQ and study skills as mediators in the AF and math achievement (teacher’s report) association.** Numbers in the figure are beta coefficients (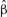 for mediational model are in parentheses). ^∗^*p* < 0.05; ^∗∗^*p* < 0.01; ^∗∗∗^*p* < 0.001.

Finally, the cognitive pathway model was tested taking this time teacher’s mathematics report as the dependent variable. The first equation of mediation assessed the relation between AF and the potential mediators. This step has been already informed above. In the second step, regressing AF on teacher’s report yielded a significant effect [*F*(2,129) = 6.62, *p* = 0.002, *R*^2^_adj_ = 0.08]. AF had a significant positive influence on math achievement informed by teachers (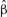 = 0.23, *p* = 0.007), after controlling for the effect of SES (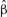 = 0.16, *p* = 0.057). On the third equation, regressing teacher’s report on AF, study skills, and IQ yielded a significant effect [*F*(4,125) = 20.47, *p* < 0.001, *R*^2^_adj_ = 0.38], with a 38% of explained variance on teacher’s math report. Study skills (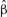 = 0.60, *p* < 0.001) and IQ (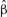 = 0.17, *p* = 0.029) showed a significant positive relationship with math achievement, even after controlling for the effect of SES (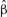 = 0.032, *p* = 0.668), whereas the relation between the independent variable (AF) and the dependent variable was non-significant (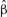 = -0.11, *p* = 0.21; see **Figure 4**). Children with higher scores on IQ and better study skills obtained better scores on teacher’s report of math achievement and, as expected, children’s attentional control had a positive effect on IQ as well as study abilities, which in turn influenced teacher’s report of math achievement. Mediation was confirmed since the bootstrapping test did not include zero in the confidence interval of neither IQ (range = 0.01–0.11) nor study skills (range = 0.24–0.49).

## Discussion

The aim of this work was to study the specific contribution of two different components of EC on the variance of children’s mathematics achievement: AF and IC. Other cognitive and socio-emotional processes were also taken into account in the mediational models explored; although it could not be applied to IC, our results suggest that the relationship between effortful attentional mechanisms and mathematics achievement is mediated in a relational pathway by peer popularity, and in an intellectual ability pathway by non-verbal intelligence and study skills. In the following sections we focus on the specific relationships found in the present study.

### EC Mechanisms and Children’s Mathematics Achievement

The first purpose of this study was to determine if the different mechanisms of EC were involved in children’s mathematics achievement. Whereas it was proved for AF, it was not the case for IC. AF was associated positively with children’s mathematics achievement, with a similar magnitude found for the standardized math battery compared to teacher’s report. Children with a higher ability to persist in ongoing tasks ignoring possible distractions as informed by their parents, showed higher mathematics abilities in comparison with their lower attentional control counterparts. These results, observed in a sample of children in the middle childhood period, are in concordance with previous research showing that children’s attentional skills positively contribute to math achievement in early and middle childhood, and adolescence (Martin et al., 1983; Martin and Holbrook, 1985; La Paro and Pianta, 2000; Duncan et al., 2007; Checa et al., 2008; Rudasill et al., 2010; Hintsanen et al., 2012). Altogether, these studies highlight that task persistence and attentional control are important for academic success in children.

However, and contrary to our expectations in line with the extensive literature involving IC (e.g., Espy et al., 2004; Blair and Razza, 2007; Clark et al., 2010; Welsh et al., 2010; Oberle and Schonert-Reichl, 2013; Allan et al., 2014), we have not observed any association between IC and children’s mathematics achievement. A possible explanation for the lack of significant results involving IC may concern the psychometric properties of this scale in our sample. IC did not reach a high internal consistency, which could have worked against Type II error (see Thompson and Vacha-Haase, 2000 for a similar interpretation), leading us to wrongly accept the null hypothesis. Since the studies that have included parental measures of EC have not reported the associations found for the specific scales that constitute this composite score, we cannot compare our results with previous literature. Another interpretation could reside, however, in the low relevance of the specific processes covered by this scale in explaining mathematics achievement. TMCQ IC was defined by its authors (Simonds and Rothbart, 2006) at the behavioral and emotional level, covering the ability for planning before acting, being cautious and careful, able to stop or slow down, and capable of inhibiting the expression of emotions when they are not appropriate; all these processes were rated by parents based on daily situations. In contrast, direct measures of IC that have been associated with mathematics ability have exposed children to specific cognitive-motor conflicts such as “go-no-go tasks,” the “day-night task,” or the size congruity effect where prepotent responses should be inhibited (e.g., Bull and Scerif, 2001; McClelland et al., 2007; Agostino et al., 2010; Liew et al., 2010; Neuenschwander et al., 2012; Fuhs and McNeil, 2013; Oberle and Schonert-Reichl, 2013; Pina et al., 2015). Nonetheless, even by using conflict direct measures with children of similar ages to ours, St Clair-Thompson and Gathercole (2006) found that a Stroop-like measure of IC was not significantly related to mathematics achievement while a stop-signal task was. The disparity of results in rather related studies draws attention to the ‘task impurity problem’ (e.g., Miyake et al., 2000) that is, the fact that IC measures also tap other constructs such as language or motor skills and therefore cannot be considered as completely equivalent (Fuhs and McNeil, 2013).

### EC Mechanisms Interacting with Relational and Intellectual Processes at School

Attentional focusing was associated positively with other school processes. In the relational area, children with higher attentional self-regulatory skills were more popular in the academic context amongst their classmates compared to children with lower self-regulation abilities. Although popularity in this study is restricted to academic activities, possibly involving rather different emotional and self-regulatory aspects than those prioritized by children when selecting others for leisure or free-play activities, our findings agree with previous research showing that specifically attentional control (e.g., Checa et al., 2008) and more broadly EC (e.g., Spinrad et al., 2006; Checa et al., 2008; Valiente et al., 2008; Zhou et al., 2010), have been associated positively with peer acceptance in the classroom.

In the intellectual sphere, AF was associated positively with non-verbal intelligence. Children with higher attentional control as informed by their parents, also showed higher general cognitive skills. Such relationship has also been found in studies that have used either questionnaires (e.g., Hintsanen et al., 2012) or direct measures of attentional control (e.g., Blair and Razza, 2007). Importantly, the behavioral results have been reinforced by the observation that higher order mechanisms of self-regulation that depends on the Executive Attentional Network (Posner and Rothbart, 2007), anatomically share brain areas related to general intelligence (Duncan et al., 2000).

At this point we should mention that differently to what it was expected, IC was not found associated neither with academic peer popularity nor intelligence. A similar explanation to that offered to explain the lack of relationship with math achievement could also be applied here.

The other intellectual ability considered in this study, that is study skills, was positively associated with AF and IC. Children with higher attentional and inhibitory self-regulatory skills faced their study with hard work, using planned strategies, and coping their tasks with self-confidence. However, taking in mind that the magnitude of the association was higher for AF compared to IC, the effortful attentional components seem to have a prominent role over the inhibitory ones on the use of proactive learning strategies at this age period. Research including academic-related skills in connection with self-regulation abilities is vast in secondary and university levels, but it is rather scarce concerning primary school years. The study by Neuenschwander et al. (2012) is one of the few that have shown a positive association of different aspects of children’ self-regulation (including a measure of EC) with learning-related behaviors in elementary school. Thus, it seems that as early as primary school-age, studying is purposeful, and requires deliberate conscious effort on the part of the students. Also, self-regulation abilities (e.g., focused attention but also initiative and goal-setting) constitute an important aspect of the studying process (Zimmerman et al., 1996; Gettinger and Seibert, 2002).

### Mediational Pathways for the Attentional Focusing-Academic Achievement Association

Following Eisenberg et al. (2005) proposal, we proved that the association AF-math achievement was partially mediated by academic peer popularity. Children with higher attentional control as informed by their parents were more popular among their classmates in the academic context, and obtained in turn higher mathematics achievement compared to children exhibiting lower attentional control. This was demonstrated for both the standard test of mathematics achievement and the teacher’s math report. Previous research on peer acceptance in early, middle childhood and adolescence had shown that children who are popular, accepted, and have positive relationships with their peers also tend to be socially well adjusted and academically more successful than those who are rejected (e.g., Wentzel, 1991; O’Neil et al., 1997; Wentzel and Caldwell, 1997; Furrer and Skinner, 2003; Ladd, 2003; Bierman, 2004; Véronneau and Vitaro, 2007; Checa et al., 2008; Chen et al., 2008; see Eisenberg et al., 2010, for a review). The relational pathway found in this work is in consonance with the previous few studies showing that self-regulation skills contribute to academic achievement in part *via* children’s social competence both concurrently (Valiente et al., 2008; Oberle and Schonert-Reichl, 2013) and predictively (e.g., Zhou et al., 2010; Valiente et al., 2011).

By comparing the two relational models (teacher’s report *versus* standard test), the observed mediational coefficients for AF when introducing peer popularity were similar for both standard test and teacher’s report. However, it is apparent that the magnitude of the relationship between peer popularity and mathematics achievement was higher for teacher’s report compared to the standard test. This finding supports the notion that teachers’ assessments include not only the knowledge of the students in a given discipline, but also other behaviors and abilities observed over an extended period of time in the context of the classroom (Graziano et al., 2007), that are probably more susceptible to the influence of socio-emotional processes developed at school (Pullis, 1985; Martin, 1989; Keogh, 2003).

The intellectual abilities pathway was also proved in that intelligence and study skills mediated the contribution of AF to mathematics achievement. Children whose parents rated them as showing better attentional control skills exhibited both higher general cognitive functioning and better learning strategies. These abilities in turn were associated with better mathematics achievement, as measured by both standard test and teacher’s report. The participation of intelligence and study skills in a wide array of academic outcomes has been already proved (e.g., Cronbach and Snow, 1977; Hunter and Hunter, 1984; Zimmerman and Martinez-Pons, 1990; Neisser et al., 1996; Purdie and Hattie, 1996; Purdie et al., 1996; McGrew et al., 1997; Jensen, 1998; Ley and Young, 1998; Keith, 1999; Pina et al., 2014). Interestingly, study skills, even though correlated moderately with intelligence, explained an independent part of the variance of mathematics achievement. As a conclusion, it seems that academic achievement does not rely uniquely on children’s general abilities but also in the effort and the quality of strategies students use in coping with their learning at school. For both general capabilities and learning skills, individual differences in attentional control play a fundamental role. As far as we know, this is the first study that shows a clear involvement of both intelligence and study skills in the relationship between effortful attention and mathematics achievement. A previous study that overlaps in part with the present one is that of Neuenschwander et al. (2012). In their study, the EC-academic achievement relationship was fully mediated by learning-related behavior. Nevertheless, EC only predicted achievement when grades were taken into account but not for standard tests.

By contrasting the two intellectual abilities mediational models tested in the current study (standard test *versus* teacher’s report), it should be noted that the relative explicative power of intelligence and study skills depends on the way mathematics achievement was measured. While non-verbal intelligence has been greatly associated with the standard test in comparison to teacher’s report, study skills has demonstrated a greater contribution to teacher’s math report. We think that the particular measurement methods we used may have inflated these associations. On the one hand, the measurement of intelligence and the administration of the standard math test were carried out by the same experimenter in a single session; on the other hand, study skills and teacher’s math reports relied on the same informant.

Finally, the relation AF-mathematics achievement was partially mediated by peer popularity irrespective of how mathematics achievement was tested. However, for the intellectual abilities pathway, the contribution of AF was fully mediated by IQ and study skills when the standard test acted as dependent variable, while it was partially mediated for teacher’s report. It means that the cognitive variables considered in this study showed a stronger explicative power on the standard test, while more room was left for children’s personality factors in explaining the variance of teacher’s math report. Again, teacher’s reports appear more susceptible to socio-emotional influences. These results lead us to conclude that although correlated, standardized tests and teacher’s reports cannot be considered equivalent forms of academic achievement. We suggest that a comprehensive approach to school performance should include both kinds of measurements.

### The Role of SES and Gender in Children’s Mathematics Achievement

A last variable included in the mediational models was SES. We found that parents’ economic and educational level was positively associated with children’s mathematics abilities as measured by standard test and teacher’s report. This study contributes to the existing literature on the relationship between family SES and academic achievement (e.g., Hart and Risley, 1995; Hoff-Ginsberg and Tardif, 1995; McLoyd, 1998; Kohl et al., 2000; Davis-Kean, 2005; Sirin, 2005; Dubow et al., 2009; Aunio and Niemivirta, 2010). Interestingly, SES was a significant predictor in the relational mediational model but not in the intellectual-abilities one. We think that this result has been produced because of the shared variance of SES with non-verbal IQ and study skills, as they were positively correlated. These relationships suggest that wealthier and more educated families would provide their children with more opportunities for learning through an environment more intellectually challenging (Davis-Kean, 2005; Pina et al., 2014).

Gender differences in mathematics achievement were also taken into account but in line with previous studies, they were not significant (e.g., Davis-Kean, 2005; Simpkins et al., 2006; Matthews et al., 2009). However, our results differ from those recently published in the last PISA Spanish report that informed of boys outperforming girls in mathematics achievement in primary school level, in agreement with the results of other studies carried out in other countries (Frome and Eccles, 1998; Jordan et al., 2006; Hintsanen et al., 2012; Neuenschwander et al., 2012). Yet others have found that gender differences in mathematics are slight, late developing and subject-specific (Leahey and Guo, 2001). The disparity of results suggests that the role of gender in the acquisition of math abilities is complex and other variables could possibly be involved in that relationship.

## Conclusion and Future Directions

In summary, the results of the present study suggest that individual differences in effortful attentional control as informed by parents, contribute to children’s mathematics achievement in middle childhood via relational and intellectual abilities. Parents observe children in a wide range of contexts and their reports constitute a valuable source of information about their children’s characteristics (Rothbart, 2011). Nevertheless, it is possible that the associations found here would have been stronger should we have used teachers as informants because their observations would have been focused on the context of the classroom. That interpretation is supported by previous research in which teacher’s reports but not parents’ reports proved associated with academic ability (e.g., Blair and Razza, 2007). Our observations of children’s study skills have also been restricted to a specific context, that is, the school. However, in the last years of primary school, students do a much more independent work out of school and parents are in a privileged position to inform about the effort their children put on school tasks at home. Since children’s behaviors vary among relevant contexts, it has been recently stressed the importance of including information from both school and home, as parents and teachers are in distinct positions that are infused with meaning as diverse agenda, investments and expectations of children in context (Fisher and Spencer, 2015).

In this study, we have considered mathematics achievement globally but in fact it is composed of a variety of skills such as arithmetic, quantitative concepts, and word-problem resolution that are dissociable. Previous research has already shown that factors such as verbal/spatial working memory, verbal ability, non-verbal intelligence, and SES have a differential involvement on different mathematics skills (Pina et al., 2014). Similarly, the variables included in the present study could have a different contribution depending on the specific mathematics skill analyzed.

The scope of this study is limited due to its correlational nature, being unable to establish a definitive causal relationship between self-regulation and academic achievement. Nevertheless, longitudinal research (e.g., Rudasill et al., 2010; Zhou et al., 2010; Rhoades et al., 2011; Valiente et al., 2011) and conceptual models (e.g., Eisenberg et al., 2010) suggest such directional effects. Importantly, our findings add to a growing body of knowledge that have identified the involvement of children’s self-regulatory abilities in academic performance, and emphasize the relevance of considering both socio-emotional and cognitive factors in the development of intervention programs in the context of school.

Finally, this work aimed to study the contribution of EC mechanisms to children’s mathematics achievement. In order to undertake this objective, we analyzed separately a relational pathway and an intellectual one, which were proved for both standard tests and teacher’s math reports. This grained approach has allowed us focusing in detail on each pathway, taking into account the kind of mathematics measurement used. Nonetheless, a limitation of the present approach is that it does not permit an analysis of the possible interactions between the relational and intellectual mediational processes and how such interactions may explain children’s mathematical performance. Further studies aimed to accomplish such broader objective are therefore of much interest.

## Conflict of Interest Statement

The authors declare that the research was conducted in the absence of any commercial or financial relationships that could be construed as a potential conflict of interest.

## Footnotes

1 Average family income in the Region of Murcia was 21,269 euros annually, retrieved from http://www.ine.es/

2 Sociogram is a common tool used at school in order to inform the teacher about social dynamics in the context of the classroom. Since it is used as part of teacher’s methodology, the explicit permission of parents is not necessary.

## References

[B1] Agostino A. Johnson J. Pascual-Leone J. (2010). Executive functions underlying multiplicative reasoning: problem type matters. *J. Exp. Child Psychol.* 105 286–305. 10.1016/j.jecp.2009.09.006 19913238

[B2] Allan N. P. Hume L. E. Allan D. M. Farrington A. L. Lonigan C. J. (2014). Relations between inhibitory control and the development of academic skills in preschool and kindergarten: a meta-analysis. *Dev. Psychol.* 50 2368–2379. 10.1037/a0037493 25069051

[B3] Alloway T. P. Alloway R. G. (2010). Investigating the predictive roles of working memory and IQ in academic attainment. *J. Exp. Child Psychol.* 106 20–29. 10.1016/j.jecp.2009.11.003 20018296

[B4] Aunio P. Niemivirta M. (2010). Predicting children’s mathematical performance in grade one by early numeracy. *Learn. Individ. Differ.* 20 427–435. 10.1016/j.lindif.2010.06.003

[B5] Baron R. M. Kenny D. A. (1986). The moderator–mediator variable distinction in social psychological research: conceptual, strategic, and statistical considerations. *J. Pers. Soc. Psychol.* 51 11–73. 10.1037/0022-3514.51.6.1173 3806354

[B6] Best J. R. Miller P. H. (2010). A developmental perspective on executive function. *Child Dev.* 81 1641–1660. 10.1111/j.1467-8624.2010.01499.x 21077853 PMC3058827

[B7] Bezanilla J. M. (2011). *Sociometria: Un Método de Investigación Psicosocial.* México City: PEI Editorial.

[B8] Bierman K. L. (2004). *Understanding and Treating Peer Rejection.* New York, NY: Guilford Press.

[B9] Birch S. Ladd G. (1997). The teacher–child relationship and children’s early school adjustment. *J. Sch. Psychol.* 35 61–79. 10.1016/S0022-4405(96)00029-5

[B10] Blair C. Razza R. P. (2007). Relating effortful control, executive function, and false belief understanding to emerging math and literacy ability in kindergarten. *Child Dev.* 78 647–663. 10.1111/j.1467-8624.2007.01019.x 17381795

[B11] Bryant A. L. Schulenberg J. Bachman J. G. O’Malley P. M. Johnston L. D. (2000). Understanding the links among school misbehavior, academic achievement, and cigarette use: a national panel study of adolescents. *Prev. Sci.* 1 71–87. 10.1023/A:1010038130788 11521961

[B12] Bull R. Scerif G. (2001). Executive functioning as a predictor of children’s mathematics ability: inhibition, switching, and working memory. *Dev. Neuropsychol.* 19 273–293. 10.1207/S15326942DN1903_3 11758669

[B13] Carnevale A. P. Smith N. Melton M. (2011). *STEM: Science Technology Engineering Mathematics.* Washington, DC: Georgetown University Center on Education and the Workforce.

[B14] Carvalho R. G. Novo R. F. (2012). Family socioeconomic status and students adaptation to school life: looking beyond grades. *Electron. J. Res. Educ. Psychol.* 10 1209–1222.

[B15] Caspi A. Wright B. R. E. Moffitt T. E. Silva P. A. (1998). Early failure in the labor market: childhood and adolescent predictors of unemployment in the transition to adulthood. *Am. Sociol. Rev.* 63 424–451. 10.2307/2657557

[B16] Checa P. Rodríguez-Bailón R. Rueda M. R. (2008). Neurocognitive and temperamental systems of self-regulation and early adolescents’ social and academic outcomes. *Mind Brain Educ.* 2 177–187. 10.1111/j.1751-228X.2008.00052.x

[B17] Chen X. Chang L. Liu H. He Y. (2008). Effects of peer group on the development of social functioning and academic achievement: a longitudinal study in Chinese children. *Child Dev.* 79 235–251. 10.1111/j.1467-8624.2007.01123.x 18366421

[B18] Clark C. A. C. Pritchard V. E. Woodward L. J. (2010). Preschool executive functioning abilities predict early mathematics achievement. *Dev. Psychol.* 46 1176–1191. 10.1037/a0019672 20822231

[B19] Credé M. Kuncel N. R. (2008). Study habits, skills, and attitudes: the third pillar supporting collegiate academic performance. *Perspect. Psychol. Sci.* 3 425–453. 10.1111/j.1745-6924.2008.00089.x 26158971

[B20] Cronbach L. J. Snow R. E. (1977). *Aptitudes and Instructional Methods.* New York, NY: Irvington.

[B21] Davis-Kean P. E. (2005). The influence of parent education and family income on child achievement: the indirect role of parental expectations and the home environment. *J. Fam. Psychol.* 19 294–304. 10.1037/0893-3200.19.2.294 15982107

[B22] Davis-Kean P. E. Schnabel Ê (1999). “The effect of socio-economic characteristics on parenting and child outcomes,” in *Proceedings of the Biennial Meeting of the Society for Research in Child Development* Albuquerque, NM.

[B23] de la Fuente J. Justicia F. J. Sander P. Elawar M. C. (2014). Personal self-regulation and regulatory teaching to predict performance and academic confidence: new evidence for the DEDEPRO mode. *Electr. J. Res. Educ. Psychol.* 12 597–620. 10.14204/ejrep.34.14031

[B24] Devine T. G. (1987). *Teaching Study Skills: A Guide for Teachers.* Boston: Allyn and Bacon.

[B25] Diamantopoulou S. Pina V. Valero-García A. V. González-Salinas C. Fuentes L. J. (2012). Validation of the spanish version of the woodcock-johnson mathematics achievement tests for children aged 6 to 13. *J. Psychoeduc. Assess.* 30 466–477. 10.1177/0734282912437531

[B26] Dubow E. F. Boxer P. Huesmann L. R. (2009). Long-term effects of parents’ education on children’s educational and occupational success: mediation by family interactions, child aggression, and teenage aspirations. *Merrill Palmer Q.* 55 224–249. 10.1353/mpq.0.0030 PMC2853053 20390050

[B27] Duckworth A. Allred K. A. (2012). “Temperament in the classroom,” in *Handbook of Temperament* eds Shiner R. L. Zentner M. (New York, NY: Guilford) 627–644.

[B28] Duncan G. J. Dowsett C. J. Claessens A. Magnuson K. Huston A. C. Klebanov P. (2007). School readiness and later achievement. *Dev. Psychol.* 43 1428–1446. 10.1037/0012-1649.43.6.1428 18020822

[B29] Duncan J. Seitz R. J. Kolodny J. Bor D. Herzog H. Ahmed A. (2000). A neural basis for general intelligence. *Science* 289 457–460. 10.1126/science.289.5478.457 10903207

[B30] DuPaul G. Rapport M. Perriello L. (1991). Teacher ratings of academic skills: the development of the academic performance rating scale. *School Psych. Rev.* 20 284–300.

[B31] Eisenberg N. Fabes R. A. Guthrie I. K. Reiser M. (2000). Dispositional emotionality and regulation: their role in predicting quality of social functioning. *J. Pers. Soc. Psychol.* 78 136–157. 10.1037/0022-3514.78.1.136 10653511

[B32] Eisenberg N. Sadovsky A. Spinrad T. L. (2005). Associations of emotion-related regulation with language skills, emotion knowledge, and academic outcomes. *New Dir. Child Adolesc. Dev.* 109 109–118. 10.1002/cd.143 16342899 PMC1361289

[B33] Eisenberg N. Valiente C. Eggum N. D. (2010). Self-regulation and school readiness. *Early Educ. Dev.* 21 681–698. 10.1080/10409289.2010.497451 21234283 PMC3018834

[B34] Else-Quest N. M. Hyde J. S. Linn M. C. (2010). Cross-national patterns of gender differences in mathematics: a meta-analysis. *Psychol. Bull.* 136 103–127. 10.1037/a0018053 20063928

[B35] Espy K. A. McDiarmid M. M. Cwik M. F. Stalets M. M. Hamby A. Senn T. (2004). The contribution of executive functions to emergent mathematic skills in preschool children. *Dev. Neuropsychol.* 26 465–486. 10.1207/s15326942dn2601_6 15276905

[B36] Fabes R. A. Eisenberg N. Jones S. Smith M. Guthrie I. Poulin R. (1999). Regulation, emotionality, and preschoolers’ socially competent peer interactions. *Child Dev.* 70 432–442. 10.1111/1467-8624.00031 10218264

[B37] Fisher L. Spencer F. (2015). Children’s Social Behaviour for Learning (SBL): reported and observed social behaviours in contexts of school and home. *Soc. Psychol. Educ.* 18 75–99. 10.1007/s11218-014-9276-4

[B38] Frome P. M. Eccles J. S. (1998). Parents’ influence on children’s achievement-related perceptions. *J. Pers. Soc. Psychol.* 74 435–452. 10.1037/0022-3514.74.2.435 9491586

[B39] Fuhs M. W. McNeil M. N. (2013). ANS acuity and mathematics ability in preschoolers from low-income homes: contributions of inhibitory control. *Dev. Sci.* 16 136–148. 10.1111/desc.12013 23278935

[B40] Furrer C. Skinner E. (2003). Sense of relatedness as a factor in children’s academic engagement and performance. *J. Educ. Psychol.* 95 148–162. 10.1037/0022-0663.95.1.148

[B41] Gettinger M. Seibert J. K. (2002). Contributions of study skills to academic competence. *School Psych. Rev.* 31 350–365.

[B42] González J. Fernández S. Pérez E. Santamaría P. (2004). *Adaptación Española de Sistema de Evaluación de la Conducta en Niños y Adolescentes: BASC.* Madrid: TEA Ediciones.

[B43] Graziano P. A. Reavis R. D. Keane S. P. Calkins S. D. (2007). The role of emotion regulation in children’s early academic success. *J. Sch. Psychol.* 45 3–19. 10.1016/j.jsp.2006.09.002 21179384 PMC3004175

[B44] Guerin D. W. Gottfried A. W. Oliver P. H. Thomas C. W. (1994). Temperament and school functioning during early adolescence. *J. Early Adolesc.* 14 200–225. 10.1177/027243169401400206

[B45] Guglielmi R. S. (2008). Native language proficiency, English literacy, academic achievement, and occupational attainment in Limited English Proficient students: a latent growth modeling perspective. *J. Educ. Psychol.* 100 322–342. 10.1037/0022-0663.100.2.322

[B46] Gumora G. Arsenio W. F. (2002). Emotionality, emotion regulation, and school performance in middle school children. *J. Sch. Psychol.* 40 395–413. 10.1016/S0022-4405(02)00108-5

[B47] Hart B. Risley T. (1995). *Meaningful Differences in the Everyday Experiences of Young American Children.* Baltimore: Brookes.

[B48] Hintsanen M. Alatupa S. Jokela M. Lipsanen J. Hintsa T. Leino M. (2012). Associations of temperament traits and mathematics grades in adolescents are dependent on the rater but independent of motivation and cognitive ability. *Learn. Individ. Differ.* 22 490–497. 10.1016/j.lindif.2012.03.006

[B49] Hoff-Ginsberg E. Tardif T. (1995). “Socioeconomic status and parenting,” in *Handbook of Parenting* Vol. 4 ed. Bornstein M. (Mahwah, NJ: Lawrence Erlbaum) 161–187.

[B50] Howse R. B. Calkins S. D. Anastopoulos A. D. Keane S. P. Shelton T. L. (2003). Regulatory contributors to children’s kindergarten achievement. *Early Educ. Dev.* 14 101–120. 10.1207/s15566935eed1401_7

[B51] Hunter J. E. Hunter R. F. (1984). Validity and utility of alternate predictors of job performance. *Psychol. Bull.* 96 72–98. 10.1037/0033-2909.96.1.72

[B52] Instituto de Evaluación. (2014). *PISA 2012. Programa Para la Evaluación Internacional de los Alumnos. Informe Español: Resultados y Contexto* Vol. 1 Madrid: Ministerio de Educación.

[B53] Jensen A. R. (1998). *The G Factor.* Westport, CT: Preager.

[B54] Jordan N. C. Kaplan D. Oláh L. N. Locuniak M. N. (2006). Number sense growth in kindergarten: a longitudinal investigation of children at risk for mathematics difficulties. *Child Dev.* 77 153–175. 10.1111/j.1467-8624.2006.00862.x 16460531

[B55] Kane M. J. Engle R. W. (2002). The role of prefrontal cortex in working-memory capacity, executive attention, and general fluid intelligence: an individual-differences perspective. *Psychon. Bull. Rev.* 9 637–671. 10.3758/BF03196323 12613671

[B56] Kaufman A. S. Kaufman N. L. (1990). *Kaufman Brief Intelligence Test.* Bloomington, MN: Pearson.

[B57] Keith T. Z. (1999). Effects of general and specific abilities on student achievement: similarities and differences across ethnic groups. *Sch. Psychol. Q.* 14 239–262. 10.1037/h0089008

[B58] Keogh B. K. (2003). *Temperament in the Classroom: Understanding Individual Differences.* Baltimore: Paul H. Brookes Publishing.

[B59] Kochanska G. Murray K. T. Coy K. C. (1997). Inhibitory control as a contributor to conscience in childhood: from toddler to early school age. *Child Dev.* 68 263–277. 10.1111/j.1467-8624.1997.tb01939.x 9180001

[B60] Kochanska G. Murray K. T. Harlan E. T. (2000). Effortful control in early childhood: continuity and change, antecedents, and implications for social development. *Dev. Psychol.* 36 220–232. 10.1037/0012-1649.36.2.220 10749079

[B61] Kohl G. O. Lengua L. J. McMahon R. J. (2000). Parent involvement in school conceptualizing multiple dimensions and their relations with family and demographic risk factors. *J. Sch. Psychol.* 38 501–523. 10.1016/S0022-4405(00)00050-9 20357900 PMC2847291

[B62] La Paro K. M. Pianta R. C. (2000). Predicting children’s competence in the early school years. A meta-analytic review. *Rev. Educ. Res.* 70 443–484. 10.3102/00346543070004443

[B63] Ladd G. W. (2003). “Probing the adaptive significance of children’s behavior and relationships in the school context: a child by environment perspective,” in *Advances in Child Development and Behavior* ed. Kail R. V. (San Diego, CA: Academic Press) 43–104. 10.1016/s0065-2407(03)31002-x 14528659

[B64] Ladd G. W. Birch S. H. Bush E. S. (1999). Children’s social and scholastic lives in kindergarten: related spheres of influence? *Child Dev.* 70 1373–1400. 10.1111/1467-8624.00101 10621962

[B65] Larson R. Richards M. H. (1991). Daily companionship in late childhood and early adolescence: changing developmental contexts. *Child Dev.* 62 284–300. 10.2307/1131003 2055123

[B66] Leahey E. Guo G. (2001). Gender differences in mathematical trajectories. *Soc. Forces* 80 713–732. 10.1353/sof.2001.0102

[B67] Ley K. Young D. B. (1998). Self-regulation behaviors in underprepared (developmental) and regular admission college students. *Contemp. Educ. Psychol.* 23 42–64. 10.1006/ceps.1997.0956 9514688

[B68] Liew J. (2012). Effortful control, executive functions, and education: bringing self-regulatory and social-emotional competencies to the table. *Child Dev. Perspect.* 6 105–111. 10.1111/j.1750-8606.2011.00196.x

[B69] Liew J. Chen Q. Hughes J. (2010). Child effortful control, teacher-student relationships, and achievement in academically at-risk children: additive and interactive effects. *Early Child. Res. Q.* 25 51–64. 10.1016/j.ecresq.2009.07.005 20161421 PMC2786182

[B70] Marjoribanks K. (2005). Family background, academic achievement, and educational aspirations as predictors of Australian young adult’s educational attainment. *Psychol. Rep.* 96 751–754. 10.2466/pr0.96.3.751-754 16050634

[B71] Martin R. P. (1989). “Activity level, distractibility, and persistence: critical characteristics in early schooling,” in *Temperament in Childhood* eds Kohnstamm G. A. Bates J. E. Rothbart M. K. (New York, NY: Wiley) 451–461.

[B72] Martin R. Drew K. Gaddis L. Moseley M. (1988). Prediction of elementary school achievement from preschool temperament: three studies. *Sch. Psychol. Rev.* 17 125–137.

[B73] Martin R. P. Holbrook J. (1985). Relationship of temperament characteristics to the academic achievement of first-grade children. *J. Psychoeduc. Assess.* 3 131–140. 10.1177/073428298500300204

[B74] Martin R. P. Nagle R. Paget K. (1983). Relationships between temperament and classroom behavior, teacher attitudes, and academic achievement. *J. Psychoeduc. Assess.* 1 377–386. 10.1177/073428298300100407

[B75] Matthews J. S. Ponitz C. C. Morrison F. J. (2009). Early gender differences in self-regulation and academic achievement. *J. Educ. Psychol.* 101 689–704. 10.1037/a0014240

[B76] McClelland M. Cameron C. E. Connor C. M. Farris C. L. Jewkes A. M. Morrison F. J. (2007). Links between behavioral regulation and preschoolers’ literacy, vocabulary, and math skills. *Dev. Psychol.* 43 947–959. 10.1037/0012-1649.43.4.947 17605527

[B77] McGrew K. S. Flanagan D. P. Keith T. Z. Vanderwood M. (1997). Beyond g: the impact of Gf–Gc specific cognitive abilities research on the future use and interpretation of intelligence tests in the schools. *Sch. Psychol. Rev.* 26 189–210.

[B78] McLoyd V. C. (1998). Socieconomic disadvantage and child development. *Am. Psychol.* 53 185–204. 10.1037/0003-066X.53.2.185 9491747

[B79] Metcalfe J. Finn B. (2013). Metacognition and control of study choice in children. *Metacogn. Learn.* 8 19–46. 10.1007/s11409-013-9094-7

[B80] Mischel W. Shoda Y. Rodriguez M. L. (1989). Delay of gratification in children. *Science* 244 933–938. 10.1126/science.2658056 2658056

[B81] Miyake A. Friedman N. P. Emerson M. J. Witzki A. H. Howerter A. (2000). The unity and diversity of executive functions and their contributions to complex “frontal lobe” tasks: a latent variable analysis. *Cogni. Psychol.* 41 49–100. 10.1006/cogp.1999.0734 10945922

[B82] Muñoz-Sandoval A. F. Woodcock R. W. McGrew K. S. Mather N. (2005). *Bateria III Woodcock- Muñoz.* Rolling Meadows, IL: Riverside.

[B83] Neisser U. Boodoo G. Bouchard T. J. Boykin A. W. Brody N. Ceci S. J. (1996). Intelligence: knowns and unknowns. *Am. Psychol.* 51 77–101. 10.1037/0003-066X.51.2.77

[B84] Neuenschwander R. Röthlisberger M. Cimeli P. Roebers C. M. (2012). How do different aspects of self-regulation predict successful adaptation to school? *J. Exp. Child Psychol.* 113 353–371. 10.1016/j.jecp.2012.07.004 22920433

[B85] Oberle E. Schonert-Reichl K. A. (2013). Relations among peer acceptance, inhibitory control, and math achievement in early adolescence. *J. Appl. Dev. Psychol.* 34 45–51. 10.1016/j.appdev.2012.09.003

[B86] O’Neil R. Welsh M. Parke R. D. Wang S. Strand C. (1997). A longitudinal assessment of the academic correlates of early peer acceptance and rejection. *J. Clin. Child Psychol.* 26 290–303. 10.1207/s15374424jccp2603_8 9292387

[B87] Pianta R. C. Stuhlman M. W. (2004). Teacher– child relationships and children’s success in the first years of school. *Sch. Psychol. Rev.* 33 444–458.

[B88] Pina V. Castillo A. Kadosh R. C. Fuentes L. J. (2015). Intentional and automatic numerical processing as predictors of mathematical abilities in primary school children. *Front. Psychol.* 6:375 10.3389/fpsyg.2015.00375 PMC4379738 25873909

[B89] Pina V. Fuentes L. J. Castillo A. Diamantopoulou S. (2014). Disentangling the effects of working memory, language, parental education, and non-verbal intelligence on children’s mathematical abilities. *Front. Psychol.* 5:415 10.3389/fpsyg.2014.00415 PMC4023045 24847306

[B90] Posner M. I. Rothbart M. K. (2000). Developing mechanisms of self-regulation. *Dev. Psychopathol.* 12 427–441. 10.1017/S0954579400003096 11014746

[B91] Posner M. I. Rothbart M. K. (2007). Research on attention networks as a model for the integration of psychological science. *Annu. Rev. Psychol.* 58 1–23. 10.1146/annurev.psych.58.110405.085516 17029565

[B92] Preacher K. J. Hayes A. F. (2008). Asymptotic and resampling strategies for assessing and comparing indirect effects in multiple mediator models. *Behav. Res. Methods* 40 879–891. 10.3758/BRM.40.3.879 18697684

[B93] Pullis M. (1985). LD Students’ temperament characteristics and their impact on decisions by resource and mainstream teachers. *Learn. Disabil. Q.* 8 109–122. 10.2307/1510413

[B94] Purdie N. Hattie J. (1996). Cultural differences in the use of strategies for self-regulated learning. *Am. Educ. Res. J.* 33 845–871. 10.3102/00028312033004845

[B95] Purdie N. Hattie J. Douglas G. (1996). Student conceptions of learning and their use of self-regulated learning strategies: a cross-cultural comparison. *J. Educ. Psychol.* 88 87–100. 10.1037/0022-0663.88.1.87

[B96] Razza R. A. Martin A. Brooks-Gunn J. (2012). The implications of early attentional regulation for school success among low-income children. *J. Appl. Dev. Psychol.* 33 311–319. 10.1016/j.appdev.2012.07.005 23243330 PMC3519429

[B97] Reynolds C. R. Kamphaus R. W. (2004). *Behavior Assessment System for Children* 2nd Edn Circle Pines, MN: AGS Publishing.

[B98] Rhoades B. L. Warren H. K. Domitrovich C. E. Greenberg M. T. (2011). Examining the link between preschool social–emotional competence and first grade academic achievement: the role of attention skills. *Early Child. Res. Q.* 26 182–191. 10.1016/j.ecresq.2010.07.003

[B99] Robbins S. B. Lauver K. Le H. Davis D. Langley R. Carlstrom A. (2004). Do psychosocial and study skill factors predict college outcomes? A meta-analysis. *Psychol. Bull.* 130 261–288. 10.1037/0033-2909.130.2.261 14979772

[B100] Rothbart M. K. (2011). *Becoming Who We Are: Temperament and Personality in Development.* New York, NY: Guilford Press.

[B101] Rothbart M. K. Bates J. E. (2006). “Temperament,” in *Handbook of Child Psychology: Social, Emotional, Personality Development* 6th Edn Vol. 1 eds Damon W. Lerner R. Eisenberg N. (New York, NY: Wiley) 99–166.

[B102] Rothbart M. K. Jones L. B. (1998). Temperament, self-regulation, and education. *Sch. Psychol. Rev.* 27 479–491.

[B103] Rothbart M. K. Sheese B. E. Posner M. I. (2007). Executive attention and effortful control: linking temperament, brain networks, and genes. *Child Dev. Perspect.* 1 2–7. 10.1111/j.1750-8606.2007.00002.x

[B104] Rudasill K. M. Gallagher K. C. White J. M. (2010). Temperamental attention and activity, classroom emotional support, and academic achievement in third grade. *J. Sch. Psychol.* 48 113–134. 10.1016/j.jsp.2009.11.002 20159222

[B105] Rueda M. R. Checa P. Combita L. M. (2012). Enhanced efficiency of the executive attention network after training in preschool children: immediate changes and effects after two months. *Dev. Cogn. Neurosci.* 2 192–204. 10.1016/j.dcn.2011.09.004 PMC6987678 22682908

[B106] Schrank F. A. Woodcock R. W. (2007). *WJ III Normative Update Compuscore and Profiles Program (Version 3.0)[Computer software]. Woodcock-Johnson III.* Rolling Meadows, IL: Riverside Publishing.

[B107] Simonds J. Kieras J. E. Rueda M. R. Rothbart M. K. (2007). Effortful control, executive attention and emotional regulation in 7 -10 year old children. *Cogn. Dev.* 22 474–488. 10.1016/j.cogdev.2007.08.009

[B108] Simonds J. Rothbart M. K. (2006). *Temperament in Middle Childhood Questionnaire.* Available at: http://www.bowdoin.edu/~sputnam/rothbart-temperament-questionnaires/

[B109] Simpkins S. D. Davis-Kean P. E. Eccles J. S. (2006). Math and science motivation: a longitudinal examination of the links between choices and beliefs. *Dev. Psychol.* 42 70–83. 10.1037/0012-1649.42.1.70 16420119

[B110] Sirin S. R. (2005). Socioeconomic status and academic achievement: a meta-analytic review of research. *Rev. Educ. Res.* 75 417–453. 10.3102/00346543075003417

[B111] Sjøberg S. (2002). Science and technology education: current challenges and possible solutions. *Innov. Sci. Technol. Educ.* 8 296–307.

[B112] Spinrad T. L. Eisenberg N. Cumberland A. Fabes R. A. Valiente C. Shepard S. A. (2006). Relation of emotion-related regulation to children’s social competence: a longitudinal study. *Emotion* 6 498–510. 10.1037/1528-3542.6.3.498 16938090 PMC1676340

[B113] St Clair-Thompson H. L. Gathercole S. E. (2006). Executive functions and achievements in school: shifting, updating, inhibition, and working memory. *Q. J. Exp. Psychol.* 59 745–759. 10.1080/17470210500162854 16707360

[B114] Taub G. E. Keith T. Z. Floyd R. G. McGrew K. S. (2008). Effects of general and broad cognitive abilities on mathematics achievement. *Sch. Psychol. Q.* 23 187–198. 10.1037/1045-3830.23.2.187

[B115] Thompson B. Vacha-Haase T. (2000). Psychometrics is datametrics: the test is not reliable. *Educ. Psychol. Meas.* 60 174–195. 10.1177/0013164400602002

[B116] Valiente C. Eisenberg N. Haugen R. Spinrad T. L. Hofer C. Liew J. (2011). Children’s effortful control and academic achievement: mediation through social functioning. *Early Educ. Dev.* 22 411–433. 10.1080/10409289.2010.505259 22573931 PMC3346258

[B117] Valiente C. Eisenberg N. Spinrad T. L. Haugen R. G. Thompson M. S. Kupfer A. (2013). Effortful control and impulsivity as concurrent and longitudinal predictors of academic achievement. *J. Early Adolesc.* 33 946–972. 10.1080/10409289.2010.505259

[B118] Valiente C. Lemery-Chalfant K. Castro K. S. (2007). Children’s effortful control and academic competence: mediation through school liking. *Merrill Palmer Q.* 53 1–25. 10.1353/mpq.2007.0006

[B119] Valiente C. Lemery-Chalfant K. Swanson J. (2010). Prediction of kindergartners’ academic achievement from their effortful control and emotionality: evidence for direct and moderated relations. *J. Educ. Psychol.* 102 550–560. 10.1037/a0018992

[B120] Valiente C. Lemery-Chalfant K. Swanson J. Reiser M. (2008). Prediction of children’s academic competence from their effortful control, relationships, and classroom participation. *J. Educ. Psychol.* 100 67–77. 10.1037/0022-0663.100.1.67 21212831 PMC3014585

[B121] Véronneau M. H. Vitaro F. (2007). Social experiences with peers and high school graduation: a review of theoretical and empirical research. *Educ. Psychol.* 27 419–445. 10.1080/01443410601104320

[B122] Vogt B. A. Finch D. M. Olson C. R. (1992). Functional heterogeneity in cingulate cortex: the anterior executive and posterior evaluative regions. *Cereb. Cortex* 2 435–443. 10.1093/cercor/2.6.435-a 1477524

[B123] Vygotsky L. S. (1978). *Mind in Society: The Development of Higher Psychological Processes.* Cambridge, MA: Harvard University Press.

[B124] Welsh J. A. Nix R. L. Blair C. Bierman K. L. Nelson K. E. (2010). The development of cognitive skills and gains in academic school readiness for children from low-income families. *J. Educ. Psychol.* 102 43–53. 10.1037/a0016738 20411025 PMC2856933

[B125] Welsh M. Parke R. D. Widaman K. O’Neil R. (2001). Linkages between children’s social and academic competence: a longitudinal analysis. *J. Sch. Psychol.* 39 463–482. 10.1016/S0022-4405(01)00084-X

[B126] Wentzel K. R. (1991). Relations between social competence and academic achievement in early adolescence. *Child Dev.* 62 1066–1078. 10.1111/j.1467-8624.1991.tb01589.x 1756656

[B127] Wentzel K. R. Caldwell K. (1997). Friendships, peer acceptance, and group membership: relations to academic achievement in middle school. *Child Dev.* 68 1198–1209. 10.1111/j.1467-8624.1997.tb01994.x 9418234

[B128] Woodcock R. W. McGrew K. S. Mather N. (2001). *Woodcock-Johnson III Tests of Achievement.* Rolling Meadows, IL: Riverside.

[B129] Zhao X. Lynch J. G. Chen Q. (2010). Reconsidering Baron and Kenny: myths and truths about mediation analysis. *J. Consum. Res.* 37 197–206. 10.1086/651257

[B130] Zhou Q. Chen S. H. Main A. (2012). Commonalities and differences in the research on children’s effortful control and executive function: a call for an integrated model of self-regulation. *Child Dev. Perspect.* 6 112–121. 10.1111/j.1750-8606.2011.00176.x

[B131] Zhou Q. Main A. Wang Y. (2010). The relations of temperamental effortful control and anger/frustration to Chinese children’s academic achievement and social adjustment: a longitudinal study. *J. Educ. Psychol.* 102 180–196. 10.1037/a0015908

[B132] Zimmerman B. J. Bonner S. Kovach R. (1996). *Developing Self-Regulated Learners: Beyond Achievement to Self-Efficacy.* Washington, DC: American Psychological Association.

[B133] Zimmerman B. J. Martinez-Pons M. (1990). Student differences in self-regulated learning: relating grade, sex, and giftedness to self-efficacy and strategy use. *J. Educ. Psychol.* 82 51–59. 10.1037/0022-0663.82.1.51

